# Impact of endoscopic ultrasonography on diagnosis of pancreatic cancer

**DOI:** 10.1007/s00535-018-1519-2

**Published:** 2018-11-07

**Authors:** Masayuki Kitano, Takeichi Yoshida, Masahiro Itonaga, Takashi Tamura, Keiichi Hatamaru, Yasunobu Yamashita

**Affiliations:** 1 Department of Gastroenterology, Wakayama Medical University School of Medicine, 811-1 Kimiidera, Wakayama-City, Wakayama 641-0012 Japan; 2 Second Department of Internal Medicine, Wakayama Medical University School of Medicine, 811-1 Kimiidera, Wakayama-City, Wakayama 641-0012 Japan

**Keywords:** Endoscopic ultrasonography, Contrast-enhanced endoscopic ultrasonography, Pancreatic cancer

## Abstract

Accumulated evidence has revealed that endoscopic ultrasonography (EUS) has had a great impact on the clinical evaluation of pancreatic cancers. EUS can provide high-resolution images of the pancreas with a quality regarded as far surpassing that achieved on transabdominal ultrasound (US), computed tomography (CT), or magnetic resonance imaging (MRI). EUS is particularly useful for the detection of small pancreatic lesions, while EUS and its related techniques such as contrast-enhanced EUS (CE-EUS), EUS elastography, and EUS-guided fine needle aspiration (EUS-FNA) are also useful in the differential diagnosis of solid or cystic pancreatic lesions and the staging (T-staging, N-staging, and M-staging) of pancreatic cancers. In the diagnosis of pancreatic lesions, CE-EUS and EUS elastography play a complementary role to conventional EUS. When sampling is performed using EUS-FNA, CE-EUS and EUS elastography provide information on the target lesions. Thus, conventional EUS, CE-EUS, EUS elastography, and EUS-FNA are essential in the clinical investigation of pancreatic cancer.

## Introduction

Pancreatic cancer is one of the leading causes of cancer-related death. It has a poor 5-year survival rate of around 8–9% [1, 2]. This is primarily because the majority of patients with pancreatic adenocarcinoma progress to either metastatic or locally advanced disease while in the asymptomatic phase. However, if pancreatic cancer is detected in the early stage (i.e., less than 2.0 cm), it has a relatively better prognosis. Therefore, accurate detection of small cancers is important for reducing the mortality rate from pancreatic cancer.

The only chance of a cure for pancreatic cancer is surgical resection. Surgery that is able to achieve clear margins and negative lymph nodes leads to a better survival rate. When evaluating the resectability of pancreatic cancer, it is important that vascular invasion, lymph node metastases, and liver metastases are appropriately evaluated.

Endoscopic ultrasonography (EUS) is an ultrasound (US) technique in which the tip of the endoscope is equipped with a high-frequency transducer. High-resolution images of the pancreas can be obtained through the esophagus, stomach, and duodenum, without the disrupting effects of intervening gas, fat, and bone. A large number of studies have demonstrated that EUS and its related techniques, including contrast-enhanced EUS (CE-EUS), EUS elastography, and EUS-guided fine needle aspiration (EUS-FNA), now play an important role in the clinical evaluation of pancreatic cancer, including the detection of small cancers, the differential diagnosis of pancreatic solid or cystic lesions, and the staging of pancreatic cancers.

In this article, the roles of EUS in the clinical investigation of pancreatic cancer, including the characterization of solid and cystic pancreatic masses and the staging of pancreatic cancers, are reviewed.

## Diagnostic techniques

### Conventional EUS

Endoscopic ultrasonography can be classed into two categories: radial and linear. Radial-type EUS provides circumferential views at right angles to the shaft of the scope, similar to those provided by CT scan. Linear format of EUS provides views in the same line or plane as the scope shaft, similar to those obtained with transabdominal US. Pancreas can be observed from 3 stations including body of the stomach, and bulb and the second portion of the duodenum. A typical endoscopic feature of the normal pancreas is a homogeneous ‘salt and pepper’ appearance. EUS has better spatial and time resolution than other imaging methods. In particular, EUS plays an important role for detection of small solid lesions and characterization of cystic lesions.

### CE-EUS

Contrast-enhanced-EUS was first reported in 1995 with an intra-arterial CO_2_ infusion [3]. After contrast agents for contrast-enhanced Doppler EUS had been improved, contrast-enhanced harmonic EUS was developed in 2008 [4]. Contrast agents consist of gas-filled microbubbles of approximately 2–5 µm in diameter, encapsulated by a phospholipid or lipid shell [5]. After the agents are administered through a peripheral vein, the microbubbles in the contrast agent receive transmitted US waves and are disrupted or stimulated to resonate, thereby producing the signal detected in the US image, which has remarkably low artifact. CE-EUS is often critical for the characterization of solid and cystic pancreatic lesions and the staging of pancreatic cancer with evaluation of lesion vascularity.

### EUS elastography

Endoscopic ultrasonography elastography for the evaluation of pancreatic tissue was first reported in 2006 [6]. The equipment can be coupled with conventional EUS without the need for additional devices. There are two types of EUS elastography, strain and shear wave. Strain elastography estimates the stiffness of the target tissue by measuring the degree of strain produced in response to compression. Shear wave elastography involves the emission of focused US from the probe to the target tissue, the so-called ‘acoustic radiation force impulse’ (ARFI), and the stiffness of the target tissue is then estimated by measuring the propagation speed of the shear wave. Only strain elastography is so far available for EUS. EUS elastography is used to characterize pancreas masses and lymph node metastases of pancreatic cancer as well as to judge the severity of chronic pancreatitis with evaluation of lesion elasticity.

### EUS-FNA

EUS-guided fine needle aspiration (EUS-FNA) has been generally used for the sampling of pancreatic tissues since it was first reported in 1992 [7]. In general, 19G–25G caliber needles are inserted under EUS guidance for the pathological diagnosis of pancreatic cancer and lymph nodes and/or hepatic metastasis of pancreatic cancer. EUS-FNA is superior to other methods such as ERCP in terms of tissue acquisition and safety. The overall complication rate of EUS-FNA is 0.82%, including complications such as pain (0.38%), bleeding (0.10%), and pancreatitis (0.4%; *n* = 8246) [8].

## Identification and characterization of solid pancreatic masses

### Conventional EUS

EUS is now regarded as the most sensitive imaging modality for the detection of pancreatic lesions. Most solid pancreatic lesions are depicted as a heterogeneous hypoechoic mass, irrespective of the pathological type. Across 22 studies covering 1170 patients, the median sensitivity of EUS for the detection of pancreatic tumors was 94% [9–30] (Table 1). The sensitivity of EUS was shown to be superior to that of computed tomography (CT; 98% vs 74%) in 19 comparative studies (*n* = 895) [9–21, 23–25, 28–30]. The sensitivity of EUS was also shown to be superior to that of transabdominal US (94% vs 67%) in four comparative studies (*n* = 259) [9, 10, 15, 30]. However, studies comparing EUS with magnetic resonance imaging (MRI) are rare.

**Table 1:** Sensitivities of conventional EUS and other imaging modalities for the detection of pancreatic masses

Number	Author	Year	References	Number of patients	EUS	CT	US	MRI
1	Rösch et al.	1991	[9]	102	99	77	67	–
2	Palazzo et al.	1993	[10]	49	91	66	64	–
3	Müller et al.	1994	[11]	33	94	69	–	83
4	Marty et al.	1995	[12]	37	92	63	–	–
5	Melzer et al.	1996	[13]	12	100	83	–	–
6	Howard et al.	1997	[14]	21	100	67	–	–
7	Sugiyama et al.	1997	[15]	73	96	86	81	–
8	Legmann et al.	1998	[16]	30	100	92	–	–
9	Gress et al.	1999	[17]	81	100	74	–	–
10	Midwinter et al.	1999	[18]	34	97	76	–	–
11	Harrison et al.	1999	[19]	19	89	68	–	–
12	Mertz et al.	2000	[20]	31	93	53	–	–
13	Rivadeneira et al.	2003	[21]	44	100	68	–	–
14	Ainsworth et al.	2003	[22]	22	87	–	–	96
15	Kitano et al.	2004	[23]	65	95	68	–	–
16	Agarwal et al.	2004	[24]	71	100	86	–	–
17	Dewitt et al.	2004	[25]	80	98	86	–	–
18	Borbath et al.	2005	[26]	59	98	–	–	88
19	Hocke et al.	2008	[27]	194	79	–	–	–
20	Jemaa et al.	2008	[28]	42	100	88	–	–
21	Sakamoto et al.	2008	[29]	36	94	50	–	–
22	Kamata et al.	2014	[30]	35	100	56	39	50
	Total number of patients			1170	1170	895	259	149
	Overall sensitivity				94	74	67	79

**Fig. 1:**
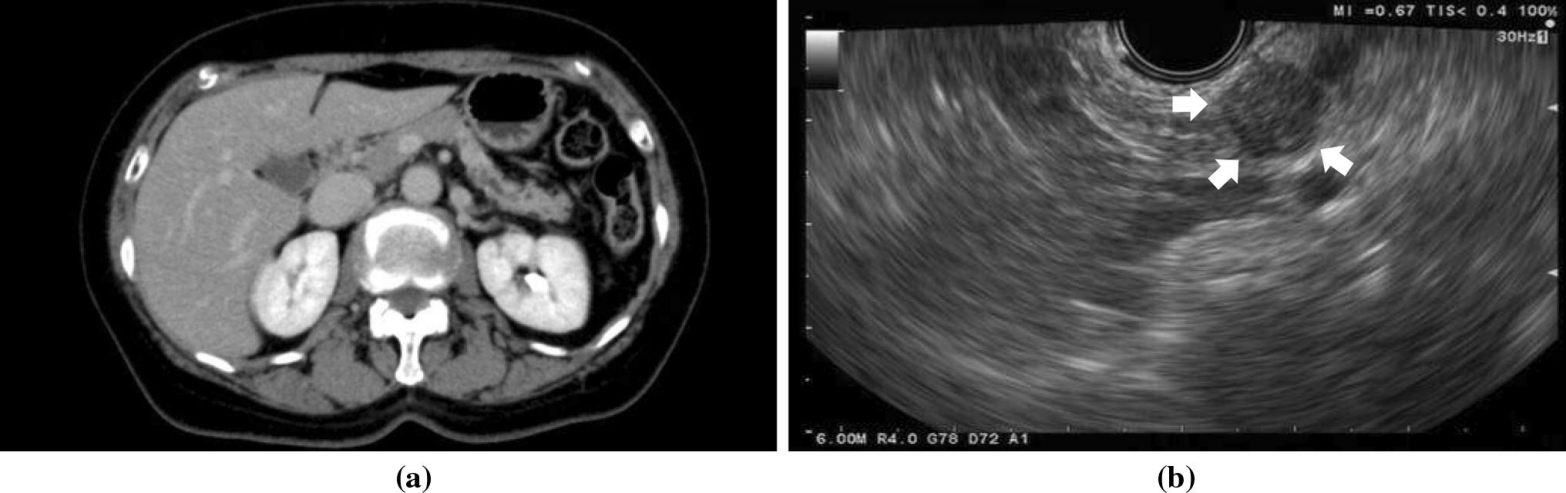
A case of small ductal carcinoma (8 mm, pancreatic body). Pancreatic mass was not detected by enhanced contrast MDCT (**a**), whereas detected clearly by endoscopic ultrasound (**b**, arrowheads)

As it has a high resolution, EUS is particularly useful for the detection of small pancreatic lesions. In a report comparing the performance of different modalities for detecting pancreatic tumors < 30 mm in diameter (*n* = 49), the sensitivities of EUS, CT, and MRI were 93%, 53%, and 67%, respectively [11]. For the detection of pancreatic tumors < 20 mm, EUS had higher sensitivity than contrast-enhanced CT (94.4 vs. 50.0%, *n* = 36) [29]. Several reports show that EUS could detect pancreatic tumors that were not identified on other modalities (Fig. 1) [24, 31–33] and a meta-analysis summarizing these four studies (*n* = 206) reported that the sensitivity of EUS for detecting pancreatic malignancy when multidetector CT findings were indeterminate was 85%, with a specificity of 58% [34]. Thus, the high sensitivity of EUS has been repeatedly confirmed. Based on the results of these studies, the clinical guideline of the Japanese Pancreas Society recommended EUS as one of the diagnostic options for patients who possibly have pancreatic cancer, alongside CT and MRI [35].

EUS is useful for the detection of small cancers. Pancreatic cancers of < 1 cm in size, accounting for 0.8% of all pancreatic cancers, has been regarded as so-called early stage cancer whose 5-year survival is reportedly 80.4% [36]. EUS can detect the small masses with a sensitivity of over 80%, which is higher than those with the other imaging methods: US (17–70%), CT (33–75%) and PET (50%) [35]. EUS reportedly can detect pancreatic tumors that were not identified on CT [23, 37].

As EUS provides high-resolution images, there has been interest in using the technique to screen asymptomatic high-risk cohorts for early cancer detection. Canto and colleagues screened 225 asymptomatic individuals considered at high risk because of hereditary and familial pancreatic cancer [38]. They blindly compared imaging studies including CT, MRI, and EUS and found that EUS was more sensitive for detecting pancreatic abnormalities (42%) than CT (11%) and MRI (33%).

### CE-EUS

In contrast to the high sensitivity of EUS for the detection of solid pancreatic masses, it is difficult to distinguish pancreatic cancer from other diseases on EUS imaging alone. Indeed, the specificity of EUS for the diagnosis of malignant pancreatic diseases is reported as 53%, with sensitivity of 95% (*n* = 115) [39].

**Fig. 2:**
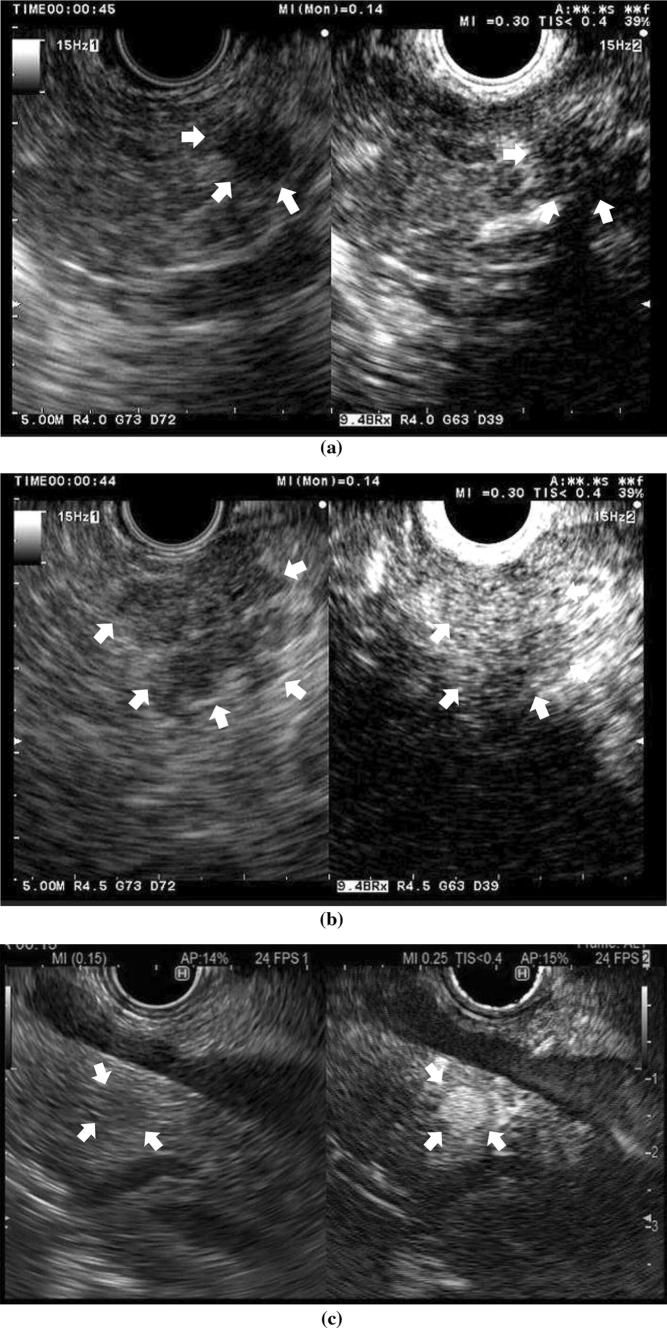
**a** A typical example of a solid lesion with hypoenhancement (a ductal carcinoma of 10 mm). Conventional EUS shows a hypoechoic area (arrowheads) at the pancreas body (left). Contrast-enhanced harmonic endoscopic ultrasonography (CH-EUS) indicates that the area is hypovascular (arrowheads) compared with the surrounding tissue (right). **b** A typical example of a solid lesion with isoenhancement (Autoimmune pancreatitis). Conventional EUS shows a hypoechoic area (arrowheads) at the pancreas head (left). CH-EUS indicates enhancement in this area similar to the surrounding tissue (arrowheads) (right). **c** A typical example of a solid lesion with hyperenhancement (a neuroendocrine tumor of 8 mm). Conventional EUS shows a hypoechoic mass (arrowheads) at the pancreas head (left). CH-EUS indicates that enhancement in the mass is higher than in the surrounding tissue (arrowheads) (right)

CE-EUS depicts most pancreatic cancers as a solid lesion with hypoenhancement (Fig. 2). CE-EUS including Doppler and harmonic modes can increase this specificity, with 20 studies (*n* = 1909) showing CE-EUS to have an estimated specificity and sensitivity of 88% and 90%, respectively [27, 29, 37, 40–56] (Table 2). In two meta-analysis, the pooled sensitivity and specificity of CE-EUS were 93–94% and 88–89%, respectively [57, 58].

**Table 2:** Diagnostic performance of contrast-enhanced EUS for solid pancreatic masses

Number	Author	Year	References	Number of patients	Sensitivity	Specificity
1	Becker et al.	2001	[41]	23	94	100
2	Hocke et al.	2008	[27]	194	92	96
3	Sakamoto et al.	2008	[29]	36	83	–
4	Dietrich et al.	2008	[42]	93	92	100
5	Fusaroli et al.	2010	[43]	90	96	98
6	Sǎftoiu et al.	2010	[44]	54	76	95
7	Napoleon et al.	2010	[45]	35	89	88
8	Seicean et al.	2010	[46]	30	80	92
9	Matsubara et al.	2011	[47]	91	96	93
10	Romagnuolo et al.	2011	[48]	21	100	73
11	Kitano et al.	2012	[38]	277	95	89
12	Imazu et al.	2012	[49]	30	100	100
13	Gheonea	2013	[50]	51	94	89
14	Lee et al.	2013	[51]	37	93	100
15	Gincul et al.	2014	[52]	100	96	94
16	Park et al.	2014	[53]	90	92	68
17	Sǎftoiu et al.	2015	[54]	167	88	100
18	Yamashita et al.	2015	[55]	147	94	71
19	Chantarojanasiri et al.	2017	[56]	136	66	63
20	Leem et al.	2018	[57]	207	82	88
	Total number of patients			1909		
	Overall				90	89
*Meta analyses*						
1	Gong et al.	2012	[58]	1139	94	89
2	He et al.	2017	[59]	1668	93	88

### EUS elastography

The overall sensitivity and specificity of EUS elastography were 93% and 63% in 15 studies (*n* = 1568) [59–73] (Table 3). On EUS elastography, the strain indicating the stiffness of target lesions may help differentiate harder pancreatic cancers from surrounding tissues. In 7 meta-analyses, the pooled sensitivity and specificity were 95–99% and 67–76%, respectively [74–80].

**Table 3:** Diagnostic performance of EUS elastography for solid pancreatic masses

Number	Author	Year	References	Number of patients	Sensitivity	Specificity
1	Janssen et al.	2007	[60]	73	100	22
2	Giovannini et al.	2009	[61]	121	92	80
3	Iglesias-Garcia et al.	2009	[62]	130	99	86
4	Itokawa et al.	2011	[63]	109	99	71
5	Sǎftoiu et al.	2012	[64]	258	88	83
6	Hocke et al.	2012	[65]	58	95	33
7	Figueiredo et al.	2012	[66]	47	90	75
8	Dawwas et al.	2012	[67]	111	100	17
9	Lee et al.	2013	[68]	35	93	86
10	Havre et al.	2014	[69]	48	67	71
11	Rustemovic et al.	2014	[70]	149	100	45
12	Kongkam et al.	2015	[71]	38	86	67
13	Opačić et al.	2015	[72]	149	98	50
14	Mayerle et al.	2016	[73]	85	77	65
15	Kim et al.	2017	[74]	157	96	96
	Total number of patients			1568		
	Overall				93	63
*Meta-analyses*						
1	Pei et al.	2012	[75]	1042	95	69
2	Mei et al.	2013	[76]	1044	95	67
3	Ying et al.	2013	[77]	893	98	69
4	Li et al.	2013	[78]	781	99	76
5	Hu et al.	2013	[79]	752	97	76
6	Xu et al.	2013	[80]	752	99	74
7	Lu et al.	2017	[81]	1537	97	67

### EUS-FNA

The sensitivities and specificities of EUS-FNA for the diagnosis of pancreatic cancer were 85–92% and 96–98%, respectively, in four meta-analyses [81–84] (Table 4). The sensitivity of EUS-FNA for pancreatic cancer exceeded 90% in patients with negative or non-diagnostic sampling from previous endoscopic retrograde cholangiopancreatography (ERCP) [85]. However, EUS-FNA cannot be applied for pancreatic cancers without forming a mass including carcinoma in situ. In those cases, ERCP-based cytology may be helpful for the diagnosis [86].

**Table 4:** Diagnostic performance of EUS-FNA

Number	Author	Year	References	Number of patients	Sensitivity	Specificity
*Meta-analyses for solid panreatic lesions*						
1	Hewitt et al.	2012	[82]	4984	85	98
2	Chen et al.	2012	[83]	1860	92	96
3	Puli et al.	2013	[84]	4766	87	96
4	Banafea et al.	2016	[85]	2761	91	97
*Meta-analyses for cystic panreatic lesions*						
1	Suzuki et al.	2014	[100]	96	65	91
2	Wang et al.	2015	[101]	1024	51	94

CE-EUS and EUS elastography may provide complementary information on the diagnosis of pancreatic cancers, in addition to the yield from EUS-FNA. CE-EUS can help to identify the EUS-FNA target, leading to a reduced requirement for repeated FNA [44, 52, 87]. The specificity of EUS-FNA may be improved when it is used with EUS elastography [70]. When CE-EUS reveals a hypovascular mass or EUS elastography reveals a hard mass in the pancreas with negative EUS-FNA findings, re-examination with EUS-FNA is recommended.

## Characterization of cystic pancreatic lesions

### Conventional EUS

Intraductal papillary mucinous neoplasms (IPMNs) and mucinous cystic neoplasms (MCNs) are cystic pancreatic lesions with a relatively high potential for malignancy, and it is difficult to exactly evaluate the malignancy of pancreatic cysts. Mural nodules within a cyst and main duct involvement suggest malignant IPMN, as indicated in several guideline [88–90]. There are limited data available on the performance of conventional EUS for the detection of mural nodules of pancreatic cystic lesions [91–96] (Table 5). Recently, Kamata et al. [93] reported that conventional EUS had a sensitivity of 97% and specificity of 40%. Harima et al. [92] reported that the sensitivity and specificity of EUS were 100% and 61%, respectively, while those of CT were 71% and 100%. The sensitivity of CT in comparison with EUS has also been reported to be as low as 24–37% [91, 94].

**Table 5:** Diagnostic performance of EUS and CT for mural nodules of IPMN

Number	Author	Year	References	Number of patients	EUS (conventioal EUS or CE-EUS)		CT	
					Sensitivity	Specificity	Sensitivity	Specificity
*Conventional EUS*								
1	Zhong et al.	2012	[92]	57	75	83	24	100
2	Harima et al.	2015	[93]	50	100	61	71	100
3	Kamata et al.	2016	[94]	70	97	40	–	–
4	Fujita et al.	2016	[95]	21	100	–	37	–
	Total number of patients			198				
	Overall				85	60	39	100
*Contrast-enhanced EUS*								
5	Kurihara et al.	2012	[96]	22	88	–	71	–
6	Yamashita et al.	2013	[97]	17	100	80	58	100
7	Harima et al.	2015	[93]	50	100	97	71	100
	Total number of patients			89				
	Overall				95	84	44	100

### CE-EUS

**Fig. 3:**
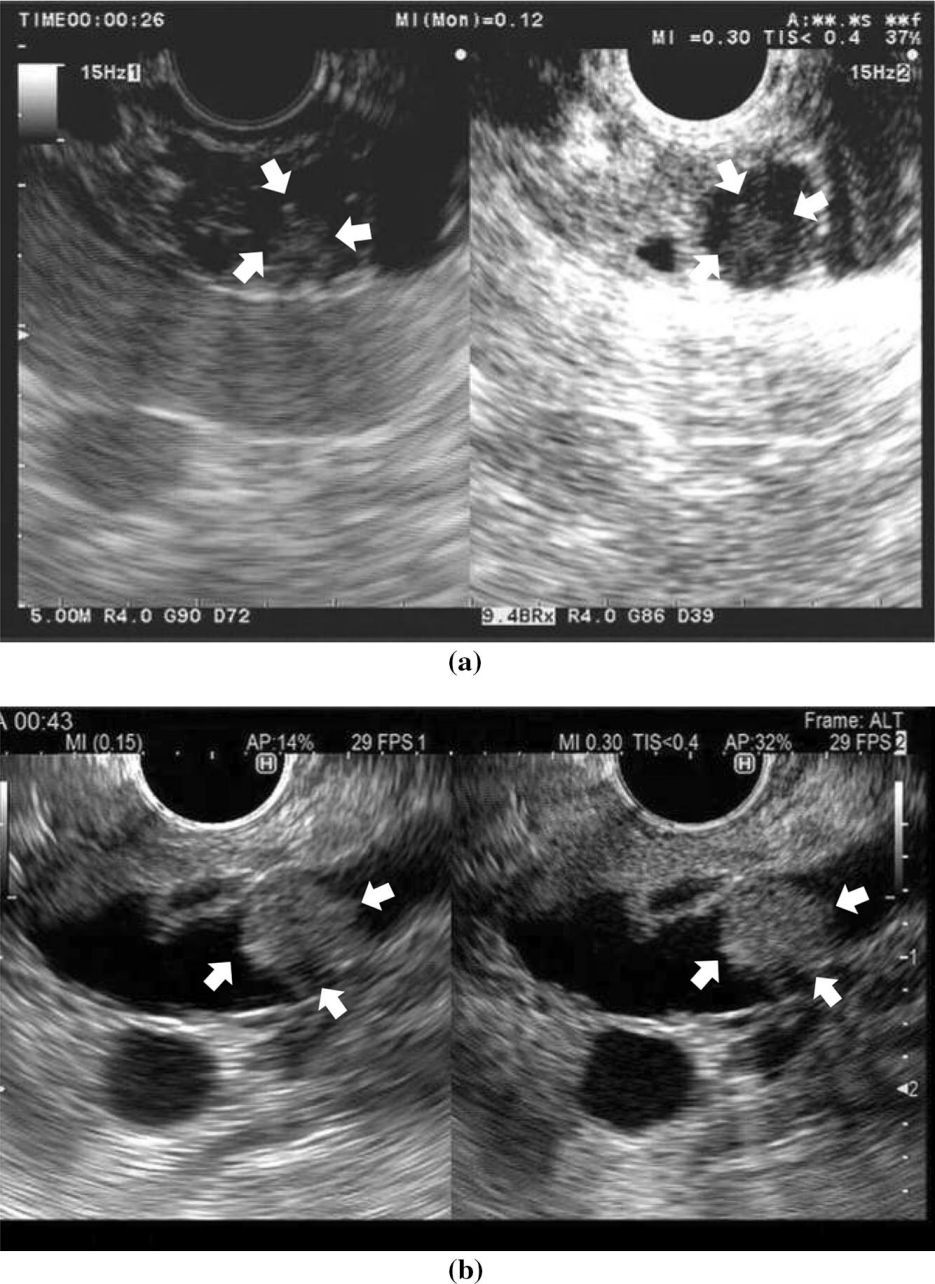
**a** A case of intraductal papillary mucinous neoplasm (IPMN) with mucous clot. Conventional EUS shows echogenic mural lesions (arrowheads) in a cyst cavity (left). Contrast-enhanced harmonic endoscopic ultrasonography (CH-EUS) shows no vascularity in the mural lesion (arrowheads) (right). **b** A case of IPMN with mural nodule. Conventional EUS shows echogenic mural lesions (arrowheads) in a cyst cavity (left). CH-EUS shows vascularity in the mural lesion (arrowheads) (right)

Mural nodules need to be distinguished from mucous clots in IPMN; however, this may sometimes be difficult on conventional EUS alone. In this respect, CE-EUS is useful for the differential diagnosis. CE-EUS depicts vascularity in mural nodules while it depicts no vascularity in mural clots (Fig. 3). Yamashita et al. [96] showed that CE-EUS can distinguish mural nodules from mucous clots with a sensitivity of 100% and a specificity of 80%, while contrast-enhanced multidetector CT achieves values of 58% and 100%, respectively. Harima et al. [92] reported sensitivity and specificity of 100% and 97% for CE-EUS, and 71% and 100% for CT.

CE-EUS is also helpful for estimating the malignant potential of IPMNs [93, 97, 98] (Table 6). Kamata et al. [93] reported that CE-EUS identified mural nodules more accurately than conventional EUS, providing sensitivity and specificity values of 97% and 75% for CE-EUS and 97% and 40% for conventional EUS. Yamamoto et al. [98] reported that the nodule/pancreatic parenchymal contrast ratio has diagnostic power for high-grade dysplasia/invasive carcinoma, with a sensitivity of 94% and specificity of 93%. Ohno et al. [97] used CE-EUS to analyze the vascularity patterns of mural nodules in IPMN and reported a sensitivity of 60% and specificity of 93%.

**Table 6:** Diagnostic performance of CE-EUS for malignant IPMN

Number	Author	Year	References	Number of patients	Contrast-enhanced EUS	
					Sensitivity	Specificity
1	Ohno et al.	2009	[98]	87	60	93
2	Kamata et al.	2016	[94]	70	97	75
3	Yamamoto et al.	2016	[99]	30	94	93
	Total number of patients			187		
	Overall				88	85

### EUS elastography

There are no reports of EUS elastography used for the diagnosis of cystic pancreatic lesions.

### EUS-FNA

In two meta-analysis, EUS-FNA-based cytology showed a sensitivity of 51% and specificity of 94% for the diagnosis of malignant pancreatic cystic lesions [99, 100]. The low sensitivity was due to factors such as sampling error. Although the carcinoembryonic antigen (CEA) level of pancreatic cyst fluid is useful for differentiating mucinous from non-mucinous pancreatic cysts, it does not correlate with the risk of malignancy [101, 102].

The DNA in pancreatic cyst fluid can also be analyzed. However, K-ras or other genetic features associated with cancer, used either alone or in combination with CEA levels, do not allow accurate differentiation of benign from malignant pancreatic cysts [103–108]. MicroRNA (miRNA) has recently been investigated and provided promising results in the differentiation of malignant from premalignant cysts; this requires further studies [109].

## T-staging of pancreatic cancer

### Conventional EUS

In the current AJCC 2010 staging criteria, T3 tumors that are potentially resectable are distinguished from T4 unresectable tumors involving celiac or superior mesenteric arteries [110]. The sensitivity and specificity of EUS for the detection of tumor vascular invasion range from 42% to 91% and 89% to 100%, respectively [17, 18, 20, 21, 111–127] (Table 7). In meta-analyses, the pooled sensitivity and specificity were 66–86% and 89–94%, respectively [128–130]. The sensitivity of EUS varies according to the target vessel. For example, the sensitivity of EUS for tumor invasion of the portal vein (PV) is over 80% [15, 131, 132], and is consistently superior to that of CT [15, 18, 115, 133] and angiography [15, 115, 131, 133]. By contrast, the sensitivity of EUS was low in comparison with that of CT in the superior mesenteric vein, superior mesenteric artery, and celiac artery [18, 20, 113, 133]. This is because it is technically difficult to provide entire images of these vessels, with this sometimes being due to obscuration by a large tumor in the uncinate or inferior portion of the pancreatic head. In general, angiography is consistently inferior to EUS and CT for assessment of vascular invasion, and has no current role in the staging of pancreatic tumors [112, 128].

**Table 7:** Diagnostic performances of EUS for the diagnosis of the vascular invasion of pancreatic cancer

Number	Author	Year	References	Number of patients	EUS	
					Sensitivity	Specificity
1	Yasuda et al.	1993	[112]	29	88	78
2	Snady et al.	1994	[113]	38	100	100
3	Gress et al.	1999	[17]	75	91	95
4	Buscail et al.	1999	[114]	32	67	100
5	Midwinter et al.	1999	[18]	30	81	80
6	Ahmad et al.	2000	[115]	89	86	71
7	Rösch et al.	2000	[116]	75	43	91
8	Shoup et al.	2000	[117]	37	20	100
9	Mertz et al.	2000	[20]	16	100	100
10	Yusoff et al.	2003	[118]	45	69	100
11	Rivadeneira et al.	2003	[21]	44	100	100
12	Soriano et al.	2004	[119]	62	42	97
13	Ramsay et al.	2004	[120]	19	56	89
14	Aslanian et al.	2005	[121]	30	50	58
15	Kulig et al.	2005	[122]	45	96	85
16	Fritsher-Ravens et al.	2005	[123]	22	86	73
17	Buchs et al.	2007	[124]	90	55	90
18	Seicean et al.	2008	[125]	30	100	54
19	Bao et al.	2008	[126]	27	80	67
20	Imazu et al.	2010	[127]	11	69	92
21	Tellez-Avila et al.	2012	[128]	50	61	90
	Total number of patients			896		
	Overall				76	86
*Meta-analyses*						
1	Nawaz et al.	2013	[129]	886	85	91
2	Li et al.	2013	[130]	368	66	94
3	Yang et al.	2014	[131]	729	72	89

### CE-EUS

There are few reports of CE-EUS for evaluation of the vascular invasion of pancreatic cancers; however, Imazu et al. [126] reported that the sensitivity and specificity for detecting PV involvement were 100% and 72.6–100%, respectively.

### EUS elastography

There are no reports of EUS elastography for T-staging of pancreatic cancer.

### EUS-FNA

There are no reports of EUS-FNA for T-staging of pancreatic cancer.

## N-staging of pancreatic cancer

### Conventional EUS

EUS is useful for the nodal staging of pancreatic cancer. In a meta-analysis (16 studies: *n* = 512), the pooled sensitivity and specificity of EUS were 69% and 81%, respectively [128]. EUS showed higher sensitivity for nodal staging than CT (58% vs 24%, eight studies, *n* = 281) [128]. Although various criteria are suggested, those mostly used are a round shape, hypoechogenicity, a smooth border, and a short axis size greater than 5 mm [10, 18]. The sensitivity of EUS is not so high because metastatic lymph nodes have variable morphologic features and partially because inflammatory changes around cancers and/or a large tumor size lead to poor images of the target lymph nodes.

### CE-EUS

Metastatic lymph nodes have been evaluated in a few patients with pancreatic cancer. Miyata et al. [134] analyzed 143 lymph nodes in 109 patients (67 patients with pancreatic cancer) with CE-EUS and found that the sensitivities and specificities of CE-EUS for the diagnosis of metastatic lymph nodes were 83% and 91%, respectively.

### EUS elastography

There have been no reports of using EUS elastography for N-staging of pancreatic cancer.

### EUS-FNA

The diagnosis of celiac lymph nodes is important from the viewpoint of evaluating surgical indications for pancreatic cancer. For the para-aortic lymph node, the sensitivity and specificity of EUS-FNA for the diagnosis of metastatic lymph nodes were 96.7% and 100%, while the sensitivity of PET-CT was only 53.3% [135].

Although conventional EUS and EUS-FNA may fail to detect small lymph node metastasis, EUS elastography is able to identify the smallest metastatic changes in tissue hardness. CE-EUS is potentially useful for target selection prior to EUS-FNA, and as suggested in the European guidelines, CE-EUS and EUS elastography provide helpful information on target lymph nodes, especially when target lymph nodes cannot be accessed with EUS-FNA or when samples for pathological evaluation are not fully obtained with EUS-FNA.

## M-staging of pancreatic cancer

### Conventional EUS

For the detection of non-nodal metastatic cancer including liver metastasis, CT and MRI are superior to EUS, because a certain portion of the right hepatic lobe located away from the upper gastrointestinal tract cannot be visualized with EUS. However, EUS can detect small hepatic lesions that would otherwise be undetected on other imaging modalities. EUS may also identify and sample ascites that may or may not have been previously detected by other imaging studies [136, 137].

### CE-EUS

Recently, Minaga et al. [138] reported that the sensitivity and specificity of EUS in the detection of left liver metastatic lesions of pancreatic cancer were 98.9% and 98.4%, respectively, while for CE-CT they were 69.7% and 73.0%, and for conventional EUS they were 97.9% and 97.6% [Minaga DDW abstract].

### EUS elastography

There are no reports of EUS elastography for M-staging of pancreatic cancer.

### EUS-FNA

Malignant ascites or liver metastases preclude surgical resections and indicate poor survival [139]. EUS-FNA has a sensitivity of 82–94% for the diagnosis of malignant ascites or liver metastasis [140–143]. Therefore, for the M-staging of pancreatic cancers, even a small quantity of ascites requires careful surveillance with EUS.

## Conclusions

Conventional EUS plays an important role in identifying pancreatic masses, particularly those of a small size. CE-EUS and EUS elastography improve the characterization of pancreatic lesions detected on EUS. EUS-FNA has high sensitivity and specificity for the detection of pancreatic cancers. CE-EUS and EUS elastography have a complementary role and assist in identifying target lesions for EUS-FNA.

## References

[1] The Editorial Board of the Cancer Statistics in Japan. Cancer Registry and Statistics. Cancer Information Service NCCJ (2018) Cancer Statics in Japan 2017. Foundation for Promotion of Cancer Research (FPCR). https://ganjoho.jp/en/professional/statistics/brochure/2017_en.html?.

[2] Noone AM, Howlader N, Krapcho M, Miller D, Brest A, Yu M, Ruhl J, Tatalovich Z, Mariotto A, Lewis DR, Chen HS, Feuer EJ, Cronin KA (eds). SEER cancer statistics review, 1975–2015. National Cancer Institute, Bethesda, MD. Based on November 2017 SEER data. 2018. https://seer.cancer.gov/csr/1975_2015/.

[3] Kato T, Tsukamoto Y, Naitoh Y (1995). Ultrasonographic and endoscopic ultrasonographic angiography in pancreatic mass lesions. Acta Radiol.

[4] Dietrich CF, Ignee A, Frey H (2005). Contrast-enhanced endoscopic ultrasound with low mechanical index: a new technique. Z Gastroenterol.

[5] Quaia E, Quaia E (2005). Classification and safety of microbubble-based contrast agents. Contrast media ultrason.

[6] Giovannini M, Hookey LC, Bories E (2006). Endoscopic ultrasound elastography: the first step towards virtual biopsy? Preliminary results in 49 patients. Endoscopy.

[7] Vilmann P, Jacobsen GK, Henriksen FW (1992). Endoscopic ultrasonography with guided fine needle aspiration biopsy in pancreatic disease. Gastrointest Endosc.

[8] Wang KX, Ben QW, Jin ZD (2011). Assessment of morbidity and mortality associated with EUS-guided FNA: a systematic review. Gastrointest Endosc.

[9] Rosch T, Lorenz R, Braig C (1991). Endoscopic ultrasound in pancreatic tumor diagnosis. Gastrointest Endosc.

[10] Palazzo L, Roseau G, Gayet B (1993). Endoscopic ultrasonography in the diagnosis and staging of pancreatic adenocarcinoma Results of a prospective study with comparison to ultrasonography and CT scan. Endoscopy.

[11] Müller MF, Meyenberger C, Bertschinger P (1994). Pancreatic tumors: evaluation with endoscopic US, CT, and MR imaging. Radiology.

[12] Marty O, Aubertin JM, Bouillot JL (1995). Prospective comparison of ultrasound endoscopy and computed tomography in the assessment of locoregional invasiveness of malignant ampullar and pancreatic tumors verified surgically. Gastroentérol Clin Biol.

[13] Melzer E, Avidan B, Heyman Z (1996). Preoperative assessment of blood vessel involvement in patients with pancreatic cancer. Isr J Med Sci.

[14] Howard TJ, Chin AC, Streib EW (1997). Value of helical computed tomography, angiography, and endoscopic ultrasound in determining resectability of periampullary carcinoma. Am J Surg.

[15] Sugiyama M, Hagi H, Atomi Y (1997). Diagnosis of portal venous invasion by pancreatobiliary carcinoma: value of endoscopic ultrasonography. Abdom Imaging.

[16] Legmann P, Vignaux O, Palazzo L (1998). Pancreatic tumors: comparison of dual-phase helical CT and endoscopic sonography. AJR.

[17] Gress FG, Hawes RH, Savides TJ (1999). Role of EUS in the preoperative staging of pancreatic cancer: a large single-center experience. Gastrointest Endosc.

[18] Midwinter MJ, Beveridge CJ, Wilsdon JB (1999). Correlation between spiral computed tomography, endoscopic ultrasonography and findings at operation in pancreatic and ampullary tumours. Br J Surg.

[19] Harrison JL, Millikan KW, Prinz RA (1999). Endoscopic ultrasound for diagnosis and staging of pancreatic tumors. Am Surg.

[20] Mertz HR, Sechopoulos P, Delbeke D (2000). EUS, PET, and CT scanning for evaluation of pancreatic adenocarcinoma. Gastrointest Endosc.

[21] Rivadeneira DE, Pochapin M, Grobmyer SR (2003). Comparison of linear array endoscopic ultrasound and helical computed tomography for the staging of periampullary malignancies. Ann Surg Oncol.

[22] Ainsworth AP, Rafaelsen SR, Wamberg PA (2003). Is there a difference in diagnostic accuracy and clinical impact between endoscopic ultrasonography and magnetic resonance cholangiopancreatography?. Endoscopy.

[23] Kitano M, Kudo M, Maekawa K (2004). Dynamic imaging of pancreatic diseases by contrast enhanced coded phase inversion harmonic ultrasonography. Gut.

[24] Agarwal B, Abu-Hamda E, Molke KL (2004). Endoscopic ultrasound-guided fine needle aspiration and multidetector spiral CT in the diagnosis of pancreatic cancer. Am J Gastroenterol.

[25] Dewitt J, Devereaux B, Chriswell M (2004). Comparison of endoscopic ultrasonography and multidetector computed tomography for detecting and staging pancreatic cancer. Ann Intern Med.

[26] Borbath I, Van Beers BE, Lonneux M (2005). Preoperative assessment of pancreatic tumors using magnetic resonance imaging, endoscopic ultrasonography, positron emission tomography and laparoscopy. Pancreatology.

[27] Hocke M, Menges M, Topalidis T (2008). Contrast-enhanced endoscopic ultrasound in discrimination between benign and malignant mediastinal and abdominal lymph nodes. J Cancer Res Clin Oncol.

[28] Jemaa Y, Houissa F, Trabelsi S (2008). Endoscopic ultrasonography versus helical CT in diagnosis and staging of pancreatic cancer. Tunis Med.

[29] Sakamoto H, Kitano M, Suetomi Y (2008). Utility of contrast-enhanced endoscopic ultrasonography for diagnosis of small pancreatic carcinomas. Ultrasound Med Biol.

[30] Kamata K, Kitano M, Kudo M (2014). Value of EUS in early detection of pancreatic ductal adenocarcinomas in patients with intraductal papillary mucinous neoplasms. Endoscopy.

[31] Wang W, Shpaner A, Krishna SG (2013). Use of EUS-FNA in diagnosing pancreatic neoplasm without a definitive mass on CT. Gastrointest Endosc.

[32] Deerenberg EB, Poley JW, Hermans JJ (2012). Role of endoscopic ultrasonography in patients suspected of pancreatic cancer with negative helical MDCT scan. Dig Surg.

[33] Meijer OLM, Weersma RK, van der Jagt EJ (2010). Endoscopic ultrasonography in suspected pancreatic malignancy and indecisive CT. Neth J Med.

[34] Krishna SG, Rao BB, Ugbarugba E (2017). Diagnostic performance of endoscopic ultrasound for detection of pancreatic malignancy following an indeterminate multidetector CT scan: a systemic review and meta-analysis. Surg Endosc.

[35] Yamaguchi K, Okusaka T, Shimizu K (2017). Clinical practice guidelines for pancreatic cancer 2016 from the Japan pancreas society a synopsis. Pancreas.

[36] Egawa S, Toma H, Ohigashi H (2012). Japan pancreatic cancer registry; 30th year anniversary: Japan pancreas society. Pancreas.

[37] Kitano M, Kudo M, Yamao K (2012). Characterization of small solid tumors in the pancreas: the value of contrast-enhanced harmonic endoscopic ultrasonography. Am J Gastroenterol.

[38] Canto MI, Hruban RH, Fishman EK (2012). Frequent detection of pancreatic lesions in asymptomatic high-risk individuals. Gastroenterology.

[39] Brand B, Pfaff T, Binmoeller KF (2000). Endoscopic ultrasound for differential diagnosis of focal pancreatic lesions, confirmed by surgery. Scand J Gastroenterol.

[40] Becker D, Strobel D, Bernatik T (2001). Echo-enhanced color- and power-doppler EUS for the discrimination between focal pancreatitis and pancreatic carcinoma. Gastrointest Endosc.

[41] Dietrich C, Ignee A, Braden B (2008). Improved differentiation of pancreatic tumors using contrast-enhanced endoscopic ultrasound. Clin Gastroenterol Hepatol.

[42] Fusaroli P, Spada A, Mancino MG (2010). Contrast harmonic echo-endoscopic ultrasound improves accuracy in diagnosis of solid pancreatic masses. Clin Gastroenterol Hepatol.

[43] Säftoiu A, Iordache S, Gheonea DI (2010). Combined contrast-enhanced power Doppler and real-time sonoelastography performed during EUS, used in the differential diagnosis of focal pancreatic masses (with videos). Gastrointest Endosc.

[44] Napoleon B, Alvarez-Sanchez MV, Gincoul R (2010). Contrast-enhanced harmonic endoscopic ultrasound in solid lesions of the pancreas: results of a pilot study. Endoscopy.

[45] Seicean A, Badea R, Stan-Iuga R (2010). Quantitative contrast-enhanced harmonic endoscopic ultrasonography for the discrimination of solid pancreatic masses. Ultraschall Med.

[46] Matsubara H, Itoh A, Kawashima H (2011). Dynamic quantitative evaluation of contrast-enhanced endoscopic ultrasonography in the diagnosis of pancreatic diseases. Pancreas.

[47] Romagnuolo J, Hoffman B, Vela S (2011). Accuracy of contrast-enhanced harmonic EUS with a second-generation perflutren lipid microsphere contrast agent (with video). Gastrointest Endosc.

[48] Imazu H, Kanazawa K, Mori N (2012). Novel quantitative perfusion analysis with contrast-enhanced harmonic EUS for differentiation of autoimmune pancreatitis from pancreatic carcinoma. Scand J Gastroenterol.

[49] Gheonea DI, Streba CT, Ciurea T (2013). Quantitative low mechanical index contrast-enhanced endoscopic ultrasound for the differential diagnosis of chronic pseudotumoral pancreatitis and pancreatic cancer. BMC Gastroenterol.

[50] Lee TY, Cheon YK, Shim CS (2013). Clinical role of contrast-enhanced harmonic endoscopic ultrasound in differentiating solid lesions of the pancreas: a single-center experience in Korea. Gut Liver.

[51] Gincul R, Palazzo M, Pujol B (2014). Contrast-harmonic endoscopic ultrasound for the diagnosis of pancreatic adenocarcinoma: a prospective multicenter trial. Endoscopy.

[52] Park JS, Kim HK, Bang BW (2014). Effectiveness of contrast-enhanced harmonic endoscopic ultrasound for the evaluation of solid pancreatic masses. World J Gastroenterol.

[53] Säftoiu A, Vilmann P, Dietrich CF (2015). Quantitative contrast-enhanced harmonic EUS in differential diagnosis of focal pancreatic masses (with videos). Gastrointest Endosc.

[54] Yamashita Y, Kato J, Ueda K (2015). Contra0st-enhanced endoscopic ultrasonography for pancreatic tumors. Biomed Res Int.

[55] Chantarojanasiri T, Hirooka Y, Kawashima H (2017). Endoscopic ultrasound in diagnosis of solid pancreatic lesions: elastography or contrast-enhanced harmonic alone versus the combination. Endosc Int Open.

[56] Leem G, Chung MJ, Park JY (2018). Clinical value of contrast-enhanced harmonic endoscopic ultrasonography in the differential diagnosis of pancreatic and gallbladder masses. Clin Endosc.

[57] Gong TT, Hu DM, Zhu Q (2012). Contrast-enhanced EUS for differential diagnosis of pancreatic mass lesions: a meta-analysis. Gastrointest Endosc.

[58] He X-K, Ding Y, Sun L-M (2017). Contrast-enhanced endoscopic ultrasound for differential diagnosis of pancreatic cancer: an updated meta-analysis. Oncotarget.

[59] Janssen J, Schlörer E, Greiner L (2007). EUS elastography of the pancreas: feasibility and pattern description of the normal pancreas, chronic pancreatitis, and focal pancreatic lesions. Gastrointest Endosc.

[60] Giovannini M, Thomas B, Erwan B (2009). Endoscopic ultrasound elastography for evaluation of lymph nodes and pancreatic masses: a multicenter study. World J Gastroenterol.

[61] Iglesias-Garcia J, Larino-Noia J, Abdulkader I (2009). EUS elastography for the characterization of solid pancreatic masses. Gastrointest Endosc.

[62] Itokawa F, Itoi T, Sofuni A (2011). EUS elastography combined with the strain ratio of tissue elasticity for diagnosis of solid pancreatic masses. J Gastroenterol.

[63] Săftoiu A, Vilmann P, Gorunescu F (2012). Efficacy of an artificial neural network-based approach to endoscopic ultrasound elastography in diagnosis of focal pancreatic masses. Clin Gastroenterol Hepatol.

[64] Hocke M, Ignee A, Dietrich CF (2012). Advanced endosonographic diagnostic tools for discrimination of focal chronic pancreatitis and pancreatic carcinoma—elastography, contrast enhanced high mechanical index (CEHMI) and low mechanical index (CELMI) endosonography in direct comparison. Z Gastroenterol.

[65] Figueiredo FAF, da Silva PM, Monges G (2012). Yield of contrast-enhanced power doppler endoscopic ultrasonography and strain ratio obtained by EUS-elastography in the diagnosis of focal pancreatic solid lesions. Endosc Ultrasound.

[66] Dawwas MF, Taha H, Leeds JS (2012). Diagnostic accuracy of quantitative EUS elastography for discriminating malignant from benign solid pancreatic masses: a prospective, single-center study. Gastrointest Endosc.

[67] Lee TH, Cho YD, Cha SW (2013). Endoscopic ultrasound elastography for the pancreas in Korea: a preliminary single center study. Clin Endosc.

[68] Havre RF, Ødegaard S, Gilja OH (2014). Characterization of solid focal pancreatic lesions using endoscopic ultrasonography with real-time elastography. Scand J Gastroenterol.

[69] Rustemovic N, Opacic D, Ostojic Z (2014). Comparison of elastography methods in patients with pancreatic masses. Endosc Ultrasound.

[70] Kongkam P, Lakananurak N, Navicharern P (2015). Combination of EUS-FNA and elastography (strain ratio) to exclude malignant solid pancreatic lesions: a prospective single-blinded study. J Gastroenterol Hepatol.

[71] Opačić D, Rustemović N, Kalauz M (2015). Endoscopic ultrasound elastography strain histograms in the evaluation of patients with pancreatic masses. World J Gastroenterol.

[72] Mayerle J, Beyer G, Simon P (2016). Prospective cohort study comparing transient EUS guided elastography to EUS-FNA for the diagnosis of solid pancreatic mass lesions. Pancreatology.

[73] Kim SY, Cho JH, Kim YJ (2017). Diagnostic efficacy of quantitative endoscopic ultrasound elastography for differentiating pancreatic disease. J Gastroenterol Hepatol.

[74] Pei Q, Zou X, Zhang X (2012). Diagnostic value of EUS elastography in differentiation of benign and malignant solid pancreatic masses: a meta-analysis. Pancreatology.

[75] Mei M, Ni J, Liu D (2013). EUS elastography for diagnosis of solid pancreatic masses: a meta-analysis. Gastrointest Endosc.

[76] Ying L, Lin X, Xie ZL (2013). Clinical utility of endoscopic ultrasound elastography for identification of malignant pancreatic masses: a meta-analysis. J Gastroenterol Hepatol.

[77] Li X, Xu W, Shi J (2013). Endoscopic ultrasound elastography for differentiating between pancreatic adenocarcinoma and inflammatory masses: a meta-analysis. World J Gastroenterol.

[78] Hu D, Gong T, Zhu Q (2013). Endoscopic ultrasound elastography for differential diagnosis of pancreatic masses: a meta-analysis. Dig Dis Sci.

[79] Xu W, Shi J, Li X (2013). Endoscopic ultrasound elastography for differentiation of benign and malignant pancreatic masses: a systemic review and meta-analysis. Eur J Gastroenterol Hepatol.

[80] Lu Y, Chen L, Li C (2017). Diagnostic utility of endoscopic ultrasonography-elastography in the evaluation of solid pancreatic masses: a meta-analysis and systematic review. Med Ultrason.

[81] Hewitt MJ, McPhail MJ, Possamai L (2012). EUS-guided FNA for diagnosis of solid pancreatic neoplasms: a meta-analysis. Gastrointest Endosc.

[82] Chen J, Yang R, Lu Y (2012). Diagnostic accuracy of endoscopic ultrasound-guided fine-needle aspiration for solid pancreatic lesion: a systematic review. J Cancer Res Clin Oncol.

[83] Puli SR, Kalva N, Bechtold ML (2013). Diagnostic accuracy of endoscopic ultrasound in pancreatic neuroendocrine tumors: a systematic review and meta analysis. World J Gastroenterol.

[84] Banafea O, Mghanga FP, Zhao J (2016). Endoscopic ultrasonography with fine-needle aspiration for histological diagnosis of solid pancreatic masses: a meta-analysis of diagnostic accuracy studies. BMC Gastroenterol.

[85] Gress F, Gottlieb K, Sherman S (2001). Endoscopic ultrasonography-guided fine-needle aspiration biopsy of suspected pancreatic cancer. Ann Intern Med.

[86] Kanno A, Masamune A, Hanada K (2018). Multicenter study of early pancreatic cancer in Japan. Pancreatology.

[87] Kitano M, Kudo M, Yamao K (2012). Characterization of small solid tumors in the pancreas: the value of contrast-enhanced harmonic endoscopic ultrasonography. Am J Gastroenterol.

[88] Tanaka M, Fernández-Del Castillo C (2012). International consensus guidelines 2012 for the management of IPMN and MCN of the pancreas. Pancreatology.

[89] Vege SS, Ziring B, Jain R (2015). American gastroenterological association institute guideline on the diagnosis and management of asymptomatic neoplastic pancreatic cysts. Gastroenterology.

[90] Del Chiaro M, Verbeke C, Salvia R (2013). European experts consensus statement on cystic tumours of the pancreas. Dig Liver Dis.

[91] Zhong N, Zhang L, Takahashi N (2012). Histologic and imaging features of mural nodules in mucinous pancreatic cysts. Clin Gastroenterol Hepatol.

[92] Harima H, Kaino S, Shinoda S (2015). Differential diagnosis of benign and malignant branch duct intraductal papillary mucinous neoplasm using contrast-enhanced endoscopic ultrasonography. World J Gastroenterol.

[93] Kamata K, Kitano M, Omoto S (2016). Contrast-enhanced harmonic endoscopic ultrasonography for differential diagnosis of pancreatic cysts. Endoscopy.

[94] Fujita M, Itoi T, Ikeuchi N (2016). Effectiveness of contrast-enhanced endoscopic ultrasound for detecting mural nodules in intraductal papillary mucinous neoplasm of the pancreas and for making therapeutic decisions. Endosc Ultrasound.

[95] Kurihara N, Kawamoto H, Kobayashi Y (2012). Vascular patterns in nodules of intraductal papillary mucinous neoplasms depicted under contrast-enhanced ultrasonography are helpful for evaluating malignant potential. Eur J Radiol.

[96] Yamashita Y, Ueda K, Itonaga H (2013). Usefulness of contrast-enhanced endoscopic sonography for discriminating mural nodules from mucous clots in intraductal papillary mucinous neoplasms a single-center prospective study. J Ultrasound Med.

[97] Ohno E, Hirooka Y, Itoh A (2009). Intraductal papillary mucinous neoplasms of the pancreas: differentiation of malignant and benign tumors by endoscopic ultrasound findings of mural nodules. Ann Surg.

[98] Yamamoto N, Kato H, Tomoda T (2016). Contrast-enhanced harmonic endoscopic ultrasonography with time-intensity curve analysis for intraductal papillary mucinous neoplasms of the pancreas. Endoscopy.

[99] Suzuki R, Thosani N, Annangi S (2014). Diagnostic yield of EUS-FNA-based cytology distinguishing malignant and benign IPMNs: a systematic review and meta-analysis. Pancreatology.

[100] Wang QX, Xiao J, Orange M (2015). EUS-guided FNA for diagnosis of pancreatic cystic lesions: a meta-analysis. Cell Physiol Biochem.

[101] Brugge WR, Lewandrowski K, Lee-Lewandrowski E (2004). Diagnosis of pancreatic cystic neoplasms: a report of the cooperative pancreatic cyst study. Gastroenterology.

[102] Othman MO, Patel M, Dabizzi E (2012). Carcino embryonic antigen and long-term follow-up of mucinous pancreatic cysts including intraductal papillary mucinous neoplasm. Dig Liver Dis.

[103] Khalid A, Zahid M, Finkelstein SD (2009). Pancreatic cyst fluid DNA analysis in evaluating pancreatic cysts: a report of the PANDA study. Gastrointest Endosc.

[104] Shen J, Brugge WR, Dimaio CJ (2009). Molecular analysis of pancreatic cyst fluid: a comparative analysis with current practice of diagnosis. Cancer.

[105] Schoedel KE, Finkelstein SD, Ohori NP (2006). K-Ras and microsatellite marker analysis of fine-needle aspirates from intraductal papillary mucinous neoplasms of the pancreas. Diagn Cytopathol.

[106] Sawhney MS, Devarajan S, O’Farrel P (2009). Comparison of carcinoembryonic antigen and molecular analysis in pancreatic cyst fluid. Gastrointest Endosc.

[107] Sreenarasimhaiah J, Lara LF, Jazrawi SF (2009). A comparative analysis of pancreas cyst fluid CEA and histology with DNA mutational analysis in the detection of mucin producing or malignant cysts. J Pancreas.

[108] Wu J, Matthaei H, Maitra A (2011). Recurrent GNAS mutations define an unexpected pathway for pancreatic cyst development. Sci Transl Med.

[109] Ryu JK, Matthaei H, Dal Molin M (2011). Elevated microRNA miR-21 levels in pancreatic cyst fluid are predictive of mucinous precursor lesions of ductal adenocarcinoma. Pancreatology.

[110] Edge S, Byrd DR, Compton CC et al. (2009) AJCC cancer staging manual | Stephen Edge | Springer [Internet]. Available from: http://www.springer.com/it/book/9780387884400#aboutBook. Accessed 8 Aug 2018.

[111] Yasuda K, Mukai H, Nakajima M (1993). Staging of pancreatic carcinoma by endoscopic ultrasonography. Endoscopy.

[112] Snady H, Bruckner H, Siegel J (1994). Endoscopic ultrasonographic criteria of vascular invasion by potentially resectable pancreatic tumors. Gastrointest Endosc.

[113] Buscall L, Pages P, Berthelemy P (1999). Role of EUS in the management of pancreatic and ampullary carcinoma: a prospective study assessing resectability and prognosis. Gastrointest Endosc.

[114] Ahmad NA, Lewis JD, Siegelman ES (2000). Role of endoscopic ultrasound and magnetic resonance imaging in the preoperative staging of pancreatic adenocarcinoma. Am J Gastroenterol.

[115] Rösch T, Dittler HJ, Strobel K (2000). Endoscopic ultrasound criteria for vascular invasion in the staging of cancer of the head of the pancreas: a blind reevaluation of videotapes. Gastrointest Endosc.

[116] Shoup M, Hodul P, Aranha GV (2000). Defining a role for endoscopic ultrasound in staging periampullary tumors. Am J Surg.

[117] Yusoff IF, Mendelson RM, Edmunds SE (2003). Preoperative assessment of pancreatic malignancy using endoscopic ultrasound. Abdom Imaging.

[118] Soriano A, Castells A, Ayuso C (2004). Preoperative staging and tumor resectability assessment of pancreatic cancer: prospective study comparing endoscopic ultrasonography, helical computed tomography, magnetic resonance imaging, and angiography. Am J Gastroenterol.

[119] Ramsay D, Marshall M, Song S (2004). Identification and staging of pancreatic tumours using computed tomography, endoscopic ultrasound and mangafodipir trisodium-enhanced magnetic resonance imaging. Australas Radiol.

[120] Aslanian H, Salem R, Lee J (2005). EUS diagnosis of vascular invasion in pancreatic cancer: surgical and histologic correlates. Am J Gastroenterol.

[121] Kulig J, Popiela T, Zaja CA (2005). The value of imaging techniques in the staging of pancreatic cancer. Surg Endosc.

[122] Fritscher-Ravens A, Knoefel WT, Krause C (2005). Three-dimensional linear endoscopic ultrasound—feasibility of a novel technique applied for the detection of vessel involvement of pancreatic masses. Am J Gastroenterol.

[123] Buchs NC, Frossard JL, Rosset A (2007). Vascular invasion in pancreatic cancer: evaluation of endoscopic ultrasonography, computed tomography, ultrasonography, and angiography. Swiss Med Wkly.

[124] Seicean A, Badea R, Mocan T (2008). Radial endoscopic ultrasonography in the preoperative staging of pancreatic cancer. J Gastrointest Liver Dis.

[125] Bao PQ, Johnson JC, Lindsey EH (2008). Endoscopic ultrasound and computed tomography predictors of pancreatic cancer resectability. J Gastrointest Surg.

[126] Imazu H, Uchiyama Y, Matsunaga K (2010). Contrast-enhanced harmonic EUS with novel ultrasonographic contrast (Sonazoid) in the preoperative T-staging for pancreaticobiliary malignancies. Scand J Gastroenterol.

[127] Tellez-Avila FI, Chavez-Tapia NC, Löpez-Arce G (2012). Vascular invasion in pancreatic cancer: predictive values for endoscopic ultrasound and computed tomography imaging. Pancreas.

[128] Nawaz H, Fan CY, Kloke J (2013). Performance characteristics of endoscopic ultrasound in the staging of pancreatic cancer: a meta-analysis. JOP.

[129] Li AE, Li BT, Ng BHK (2013). Diagnostic accuracy of imaging modalities in the evaluation of vascular invasion in pancreatic adenocarcinoma: a meta-analysis. World J Oncol.

[130] Yang R, Lu M, Qian X (2014). Diagnostic accuracy of EUS and CT of vascular invasion in pancreatic cancer: a systematic review. J Cancer Res Clin Oncol.

[131] Brugge WR, Lee MJ, Kelsey PB (1996). The use of EUS to diagnose malignant portal venous system invasion by pancreatic cancer. Gastrointest Endosc.

[132] Rösch T, Braig C, Gain T (1992). Staging of pancreatic and ampullary carcinoma by endoscopic ultrasonography. Comparison with conventional sonography, computed tomography, and angiography. Gastroenterology.

[133] Rosch T, Dittler HJ, Lorenz R (1992). The endosonographic staging of pancreatic carcinoma. Dtsch Med Wochenschr.

[134] Miyata T, Kitano M, Omoto S (2016). Contrast-enhanced harmonic endoscopic ultrasonography for assessment of lymph node metastases in pancreatobiliary carcinoma. World J Gastroenterol.

[135] Kurita A, Kodama Y, Nakamoto Y (2016). Impact of EUS-FNA for preoperative para-aortic lymph node staging in patients with pancreatobiliary cancer. Gastrointest Endosc.

[136] Chang KJ, Albers CG, Nguyen P (1995). Endoscopic ultrasound-guided fine needle aspiration of pleural and ascitic fluid. Am J Gastroenterol.

[137] Nguyen PT, Chang KJ (2001). EUS in the detection of ascites and EUS-guided paracentesis. Gastrointest Endosc.

[138] Minaga K, Kitano M, Takenaka M (2017). Improved diagnosis of liver metastases using Kupffer-phase image of contrast-enhanced harmonic EUS in patients with pancreatic cancer. Gastrointest Endosc.

[139] DeWitt J, Yu M, Al-Haddad MA (2010). Survival in patients with pancreatic cancer after the diagnosis of malignant ascites or liver metastases by EUS-FNA. Gastrointest Endosc.

[140] Nguyen P, Feng JC, Chang KJ (1999). Endoscopic ultrasound (EUS) and EUS-guided fine-needle aspiration (FNA) of liver lesions. Gastrointest Endosc.

[141] Hollerbach S, Willert J, Topalidis T (2003). Endoscopic ultrasound-guided fine-needle aspiration biopsy of liver lesions: histological and cytological assessment. Endoscopy.

[142] TenBerge J, Hoffman BJ, Hawes RH (2002). EUS-guided fine needle aspiration of the liver: indications, yield, and safety based on an international survey of 167 cases. Gastrointest Endosc.

[143] DeWitt J, LeBlanc J, McHenry L (2003). Endoscopic ultrasound-guided fine needle aspiration cytology of solid liver lesions: a large single-center experience. Am J Gastroenterol.

